# Changes in higher-order aberrations: toric implantable collamer lens implantation versus ICL combined with limbal relaxing incision

**DOI:** 10.3389/fopht.2026.1812066

**Published:** 2026-07-03

**Authors:** Zhichao Liu, Caiyuan Liu, Ruixia Li, Lin Zheng, Shangkun Ou

**Affiliations:** 1 Xiamen Eye Center and Eye Institute of Xiamen University, School of Medicine, Department of Refractive Surgery, Xiamen, Fujian, China; 2 Xiamen Clinical Research Center for Eye Diseases, Xiamen, Fujian, China; 3 The Affiliated Hospital of Guizhou Medical University, Department of Ophthalmology, Guiyang, Guizhou, China

**Keywords:** astigmatism, higher-order aberrations, implantable collamer lens, limbal relaxing incision, vector analysis

## Abstract

**Purpose:**

To compare the safety, efficacy and changes in corneal aberrations between Implantable Collamer Lens (ICL) combined with Limbal Relaxing Incision (LRI) and Toric ICL (TICL) implantation for moderate astigmatism (1.00-1.75 D).

**Methods:**

A retrospective study included 30 subjects (46 eyes) assigned to ICL combined with LRI (23 eyes) or TICL (23 eyes) group. Uncorrected distance visual acuity (UDVA), objective refraction, astigmatism vector analysis were assessed preoperatively and at 1 week, 1 month and 3 months postoperatively; corneal aberrations were compared preoperatively and 3 months postoperatively.

**Results:**

Both groups showed comparable outcomes in visual acuity, refraction postoperatively. Surgically Induced Astigmatism (SIA) showed a decreasing trend in the LRI group (1.07 to 0.92, P = 0.044), while it remained stable in the TICL group (1.11 to 1.20, P = 0.507). Total corneal wavefront aberrations, spherical aberration and horizontal coma postoperatively showed reduction in LRI group, whereas slightly increased in the TICL group (all P< 0.05).

**Conclusion:**

Both ICL combined with LRI and TICL implantation achieve good efficacy in correcting moderate astigmatism. The TICL group showed better postoperative astigmatism stability. The LRI group demonstrated reductions in several corneal aberration parameters compared with the TICL group.

## 1 Introduction

Currently, Intraocular Lens Implantation has gained increasing popularity and acceptance in modern refractive surgeries. This procedure provides a wider range of refractive correction without corneal ablation, thereby satisfying the requirements of patients with high myopia (1–4).

Implantable collamer lens (EVO Visian ICL, STAAR Surgical, Lake Forest, CA, USA) provides myopia correction over a range from -0.50 to -18.00 diopters (D); simultaneously, Toric ICL corrects astigmatism ranging from 0.50 to 6.00 D, and its efficacy has been well validated in previous studies (5, 6). However, the application of Toric ICL is also associated with the risk of lens rotation (7), and also has drawbacks such as long waiting times and relatively high costs. As a compensatory strategy, limbal Relaxing Incision (LRI) corrects astigmatism through single or paired relaxing incisions on the steep meridian of the cornea, suitable for astigmatism correction up to 2.00 D (8). Accumulating evidence indicates the satisfactory clinical outcomes of ICL combined with LRI for correcting low-to-moderate astigmatism (5). Given its advantages in cost-effectiveness and convenience, this combined approach may serve as an alternative to TICL implantation.

Previous studies compared these two surgical approaches for correcting low-to-moderate astigmatism (9) showing that both protocols achieved comparable outcomes in improving visual acuity and refractive outcomes. Nonetheless, as an essential indicator of postoperative visual quality, higher-order aberrations (HOA) also deserve clinical attention, especially for the refinement of these specific surgical modalities. It has been well established in the literature that ICL surgeries induce changes in higher-order aberrations (10). Regarding the impact of LRIs, while its combinations with cataract surgery have been well-documented to induce an elevation in HOAs (11), investigations following the joint ICL and LRI approach remain highly limited. Therefore, for a better evaluation of the two surgical approaches, we believe it is necessary to conduct a comparative study on HOA changes between ICL combined with LRI and Toric ICL implantation. Considering the target population of Toric ICLs and the correction limit of LRI technique, as well as to better control for confounding variables, we restricted this study to patients with moderate astigmatism (preoperative cylinder: 1.00–1.75 D).

## 2 Materials and methods

### 2.1 Subjects

This was a retrospective observational study. A total of 30 patients (46 eyes) with myopia and moderate astigmatism who underwent ICL implantation at Xiamen Eye Center and Eye Institute of Xiamen University between November 2023 and June 2025 were enrolled, all surgeries were performed by one experienced surgeon (ZL). Patients were divided into two groups according to the surgical procedure: TICL implantation group and ICL + LRI group. The study protocol was approved by the Clinical Research Medical Ethics Committee of Xiamen Eye Center (Approval No.: XMYKZX-LW-2026-004) and adhered to the tenets of the Declaration of Helsinki. Prior to the use of clinical data for analysis and publication, written informed consent was obtained from all patients.

Inclusion criteria: Myopia ≤ 18.00 D; 1.00 ≤ Astigmatism ≤ 1.75 D; Age between 18 and 38 years; Endothelial cell count > 2000/mm²; Anterior chamber depth ≥ 2.8 mm.

Exclusion criteria: Preoperative presence of ocular diseases such as glaucoma, cataract, and keratoconus; Narrow anterior chamber angles or related anatomical structural abnormalities; Active inflammation of the ocular surface; Presence of presbyopia.

### 2.2 Surgical procedure for the TICL group

Preoperatively, sufficient mydriasis was achieved with compound tropicamide eye drops. The surgeon manually marked the axis of astigmatism at the slit lamp, and surface anesthesia was administered with Proparacaine Hydrochloride (Alcon Inc., Fort Worth, TX, USA). The operative eye was disinfected with povidone-iodine, and the implantable lens was loaded into the implantation injector. A 2.8 mm main incision was made by gemstone scalpels (Alcon Inc., Fort Worth, TX, USA) at the limbus, with its location strategically determined based on the predicted residual astigmatism (if the intended astigmatism correction of Toric lens fully matched the manifest cylinder, the incision was made horizontally; conversely, if under-correction was anticipated, the incision was made on the steep meridian), side port incisions were not applied in surgeries. After the ICL was implanted into the anterior chamber using an injector cartridge, viscoelastic agent (Shanghai Qisheng Biological Preparation Co., Ltd., Shanghai, China) was injected to maintain the anterior chamber. A lens positioning hook was utilized to place the four haptics into the ciliary sulcus of the posterior chamber, and the lens axis was adjusted to align with the limbal astigmatism marking. The viscoelastic agent was removed with a balanced salt solution. Following the hydration of all incisions, the surgeon made a final verification of lens centration and positioning. Postoperatively, antibiotics, artificial tears and corticosteroid eye drops were administered for 4 weeks (antibiotic stopped and corticosteroid decreased to 2 times a day at 1 week).

### 2.3 Surgical procedure for the ICL + LRI group

Preoperatively, the length, position and ablation depth of LRIs were obtained from the LRI calculation website (www.lricalculator.com). The surgeon’s surgery induced astigmatism was considered as 0.5 D. The horizontal axis was marked at 0° and 180° of the operative eyes at the slit lamp. During the surgery, the main incision location was marked on the steep meridian, and the midpoints of the limbal relaxing incisions were marked on the limbus opposite the main incision. A limbal relaxing cup and a corneal depth-controlled knife (MANI, Inc., Utsunomiya, Japan) were employed to construct the limbal relaxing incisions. The remaining surgical steps were performed in an identical manner to those in the TICL group.

### 2.4 Preoperative baseline and postoperative follow-up measures

All patients were evaluated preoperatively and followed up at 1 week, 1 month, and 3 months postoperatively. The parameters measured at each visit included uncorrected distance visual acuity, objective refraction, and intraocular pressure. Corneal topography was performed using the Scheimpflug analysis system Pentacam HR (Oculus, Wetzlar, Germany). The following parameters were collected: horizontal vault height, central corneal thickness, anterior chamber depth and anterior △K, K1, K2. Zernike analysis was performed with a maximum area of 6.00 mm and a corneal refractive index of 1.376. Wavefront aberration parameters included the root mean square (RMS) of higher-order aberrations (HOA) and the RMS of total wavefront aberrations. All higher-order aberrations from the 3rd to the 4th order were recorded, all wavefront aberration data were collected from three regions: anterior corneal surface, posterior corneal surface, and total cornea.

### 2.5 Vector analysis

Vector analysis was used to evaluate the astigmatism correction outcomes. Surgically Induced Astigmatism (SIA) was measured according to the Alpins-Goggins method (12). SIA = Preoperative cylinder - Postoperative cylinder, i.e., the vector difference between the preoperative and postoperative measured cylinders. Target-Induced Astigmatism (TIA) refers to the expected vector of cylinder change. Additionally, the Correction Index (CI) was calculated to assess the matching degree between the actual correction power and the target correction power, using the formula: CI = |SIA|/|TIA|. Ideally, the CI value is close to 1; CI > 1.0 D indicates overcorrection, and CI< 1.0 D indicates undercorrection.

### 2.6 Statistical analysis

Statistical analysis was performed using SPSS 21.0 software (IBM Corp. Al Monk, NY, USA). Independent samples t-test and Mann-Whitney U Test were used to compare data between groups depending on data distribution. Paired t-test, Repeated Measures ANOVA, Friedman Test was used to compare intra-group parameters at different time points. Quantitative data were expressed as mean ± standard deviation. Categorical data were analyzed using the chi-square test. A P-value< 0.05 was considered statistically significant.

## 3 Results

A total of 30 patients (46 eyes) were included in the study, with a mean age of 26.67 ± 3.42 years (range: 19 to 36 years), and 56.67% were female. The ICL + LRI group included 16 patients (23 eyes), and the TICL group included 14 patients (23 eyes).

### 3.1 Preoperative baseline parameters

As shown in **Table 1**: In the TICL group, the spherical equivalent was -8.50 D (IQR: -9.375 D, -7.75 D; Range: -10.00 D to -2.75 D), and the astigmatism was -1.38 ± 0.22 D (-1.75 to -1.00 D). In the ICL + LRI group, the spherical equivalent was -7.75 D (IQR: -9.12D, -6.75 D; Range: -9.875 D to -3.00 D), and the astigmatism was -1.28 ± 0.20 D (-1.75 to -1.00 D) preoperatively. There were no statistically significant differences in spherical equivalent or cylinder between the two groups. Additionally, no significant differences were observed in corrected distance visual acuity measured in LogMAR, anterior chamber depth, white-to-white (WTW) distance, or IOP between the two groups. The central corneal thickness (CCT) in the TICL group was higher than that in the ICL + LRI group (P = 0.002). The anterior corneal astigmatism was also found higher in TICL group than in LRI group (P = 0.001).

**Table 1:** Patient baseline characteristics.

Parameter	Group		P value
	LRI+ICL	TICL	
Gender (male/female)	5/11	8/6	
Age (y)	26.62 ± 4.16	27.14 ± 4.17	0.955
CDVA (logMAR)	0 (0,0)	0 (0,0)	0.639
MRSE (D)	-7.75 (-9.12, -6.75)	-8.50 (-9.37, -7.75)	0.132
Cylinder (D)	-1.28 ± 0.20	-1.38 ± 0.22	0.129
IOP (mmHg)	14.37 ± 2.10	15.70 (13.30,18.10)	0.160
CCT (μm)	511.52 ± 25.62	536.91 ± 27.27	0.002
ACD (mm)	3.21 ± 0.24	3.07 (2.99,3.21)	0.141
Corneal astigmatism (D)	1.39 ± 0.40	1.82 ± 0.44	0.001

Continuous data with normal distribution are presented as mean ± SD and analyzed by Student’s t test; Continuous data without normal distribution are presented as median (IQR) and analyzed by the Wilcoxon rank sum test; Categorical data are presented as n (%) and analyzed by the chi-squared test; CDVA, corrected distance visual acuity; logMAR, logarithm of the minimum angle of resolution; MRSE, mean refractive spherical equivalent; IOP, intraocular pressure; CCT, Central Corneal Thickness; ACD, anterior chamber depth.

Bold values indicate statistically significant differences (P < 0.05).

### 3.2 Postoperative visual outcome and refraction

**Figure 1:**
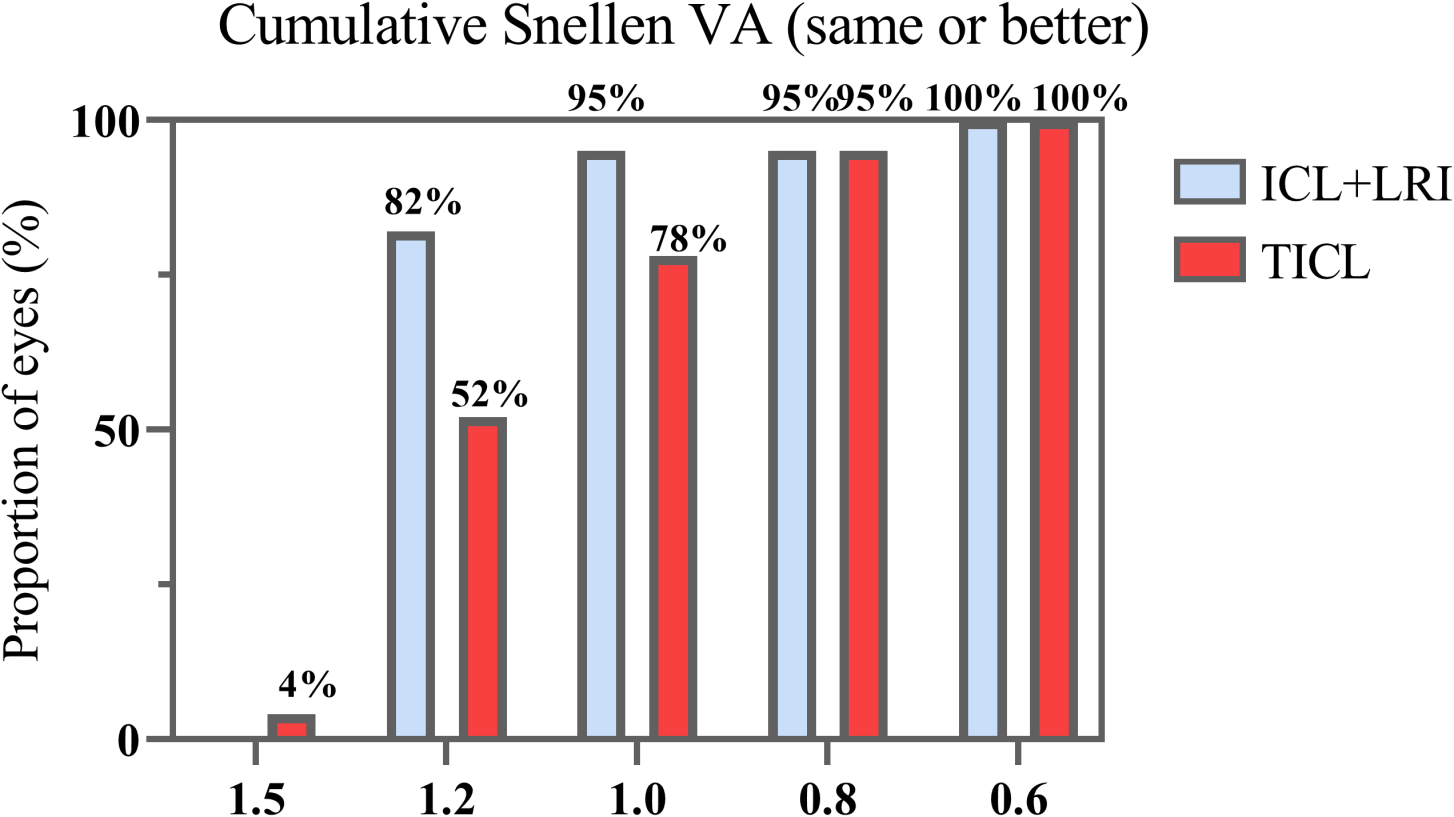
Proportions of eyes with different postoperative Snellen visual acuity levels.

**Figure 2:**
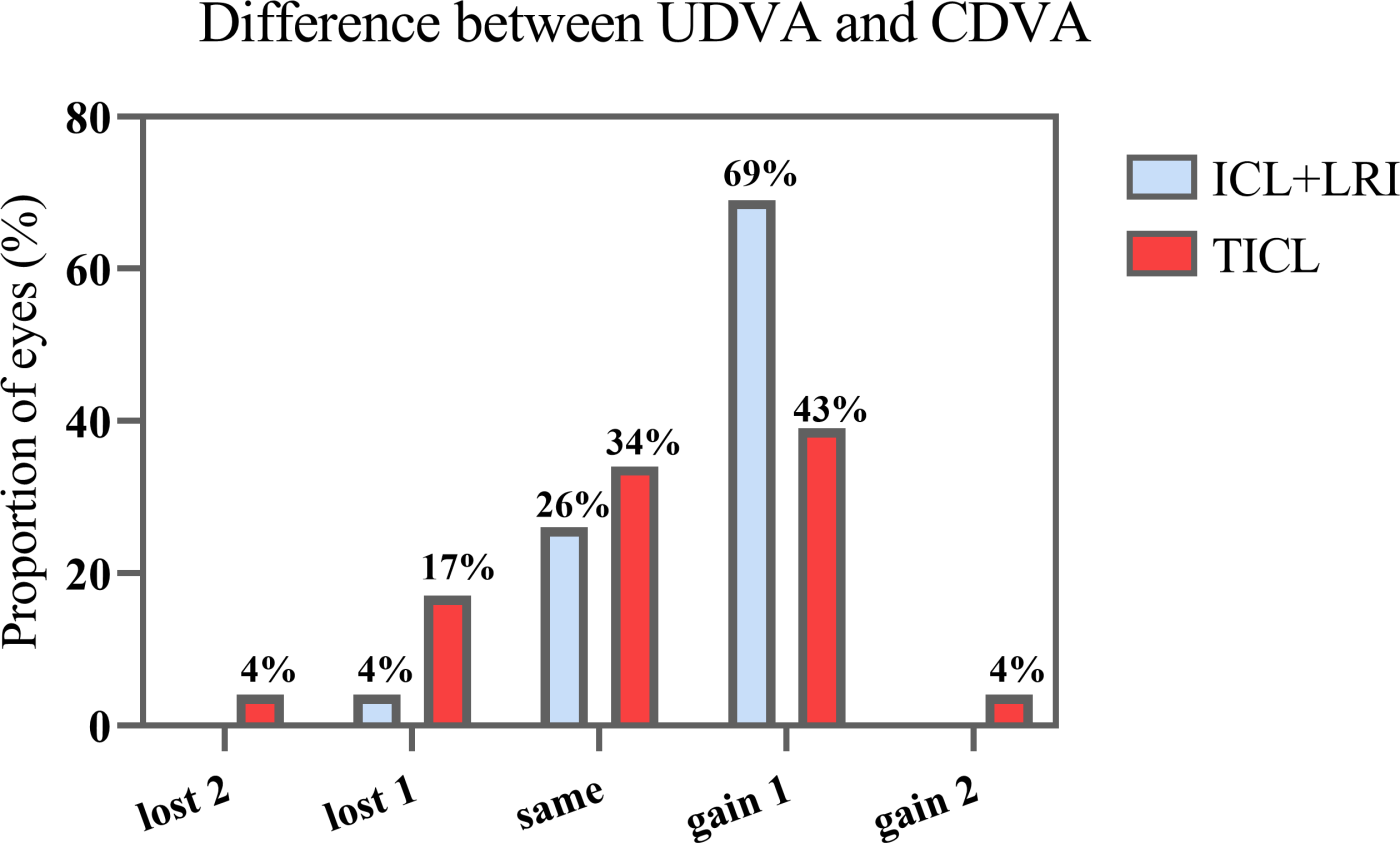
Difference between postoperative UDVA and preoperative CDVA.

Postoperative visual acuity, spherical equivalent, and cylinder are shown in **Table 2**, **Figures 1**, **2**. The uncorrected distance visual acuity of both the LRI group and the TICL group was significantly improved compared with the preoperative level, with no statistically significant difference between the groups. In both groups, 95% of the patients had UDVA of not less than 0.8 at 3 months postoperatively. The proportion of patients with visual acuity of not less than 1.0 in the LRI group was higher than that in the TICL group at 3 months postoperatively (LRI: 95% vs TICL: 78%); The UDVA (logMAR) of the LRI group was slightly better than that of the TICL group at the 3-month follow-up (P = 0.048). One case in the ICL + LRI group and 4 cases in the TICL group had postoperative UDVA 1 line lower than the preoperative CDVA, and 1 case in the TICL group had postoperative UDVA 2 lines lower than the preoperative CDVA. Both surgical procedures effectively corrected astigmatism, with no significant difference observed between the groups within 3 months postoperatively. The IOP in the TICL group was higher than that in the LRI group at 1 week postoperatively (P< 0.001), but no significant difference was observed in subsequent follow-ups. At 3 months postoperatively, the efficacy index was 1.13 ± 0.11 in the ICL + LRI group and 1.05 ± 0.20 in the TICL group, with no statistically significant difference between the groups (P = 0.09).

**Table 2:** Postoperative visual, refractive, and topographic outcomes.

Parameter	Group		P value
	LRI+ICL	TICL	
UDVA			
1 week	-0.05 ± 0.04	-0.08 (-0.08,0.00)	0.438
1 month	-0.07 ± 0.06	-0.08 (-0.08,0.00)	0.320
3 months	-0.08 (-0.08, -0.08)	-0.02 ± 0.09	0.048
MRSE			
1 week	0.18 ± 0.42	0.11 ± 0.42	0.571
1 month	0.22 ± 0.38	0.13 ± 0.44	0.481
3 months	0.13 ± 0.47	-0.02 ± 0.42	0.252
Cylinder			
1 week	-0.31 ± 0.19	-0.41 ± 0.27	0.159
1 month	-0.38 ± 0.21	-0.34 ± 0.27	0.544
3 months	-0.44 ± 0.24	-0.44 ± 0.27	1.000
IOP			
1 week	13.6 (12.2,15.0)	17.3 ± 3.00	< 0.001
1 month	14.72 ± 2.07	13.60 ± 2.40	0.099
3 months	13.7 ± 2.50	13.73 ± 2.20	0.965
TIA(D)	-1.28 ± 0.20	-1.38 ± 0.22	0.132
SIA(D)			
1 week	1.07 ± 0.30	1.11 ± 0.43	0.669
1 month	1.02 ± 0.31	1.19 ± 0.45	0.155
3 months	0.92 ± 0.20	1.20 ± 0.42	0.007
CI			
1 week	0.84 (0.61, 0.97)	0.80 ± 0.29	0.700
1 month	0.80 ± 0.24	0.85 ± 0.27	0.543
3 months	0.68 (0.62, 0.82)	0.87 ± 0.28	0.097
Vault (μm)	240.00 (170.00,370.00)	340.00 (190.00,480.00)	0.262
ACD (mm)	3.05 ± 0.23	2.93 (2.83,3.03)	0.132
Corneal astigmatism (D)	-0.47 ± 0.41	0.10 (-0.10,0.30)	< 0.001

UDVA, uncorrected distance visual acuity; MRSE, mean refractive spherical equivalent; IOP, intraocular pressure; TIA, target-induced astigmatism; SIA, surgically induced astigmatism; CI, correction index; ACD, anterior chamber depth.

Bold values indicate statistically significant differences (P < 0.05).

### 3.3 Astigmatism vector analysis

**Figure 3:**
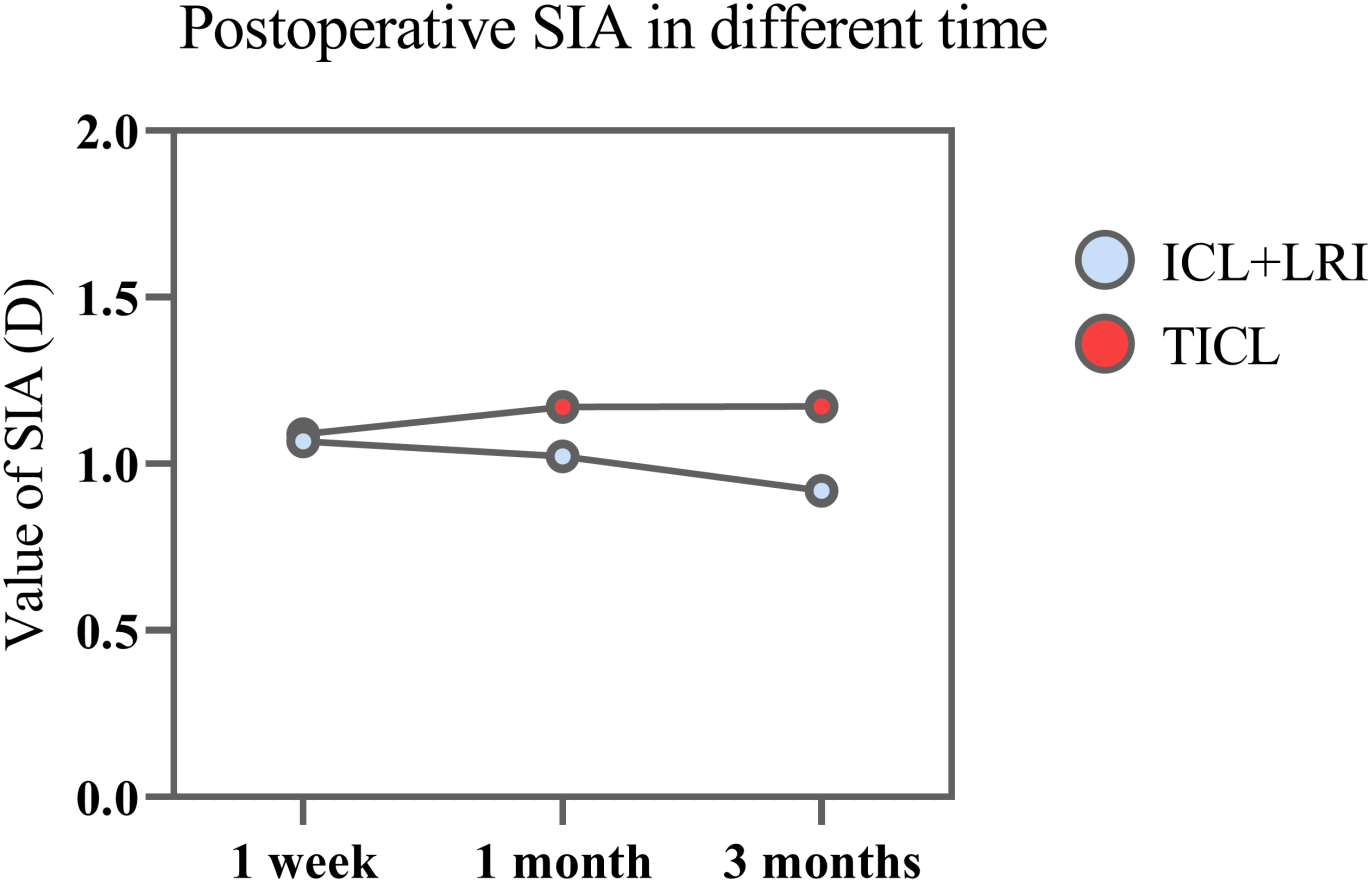
Postoperative surgically induced astigmatism (SIA) at different follow-up time points.

**Figure 4:**
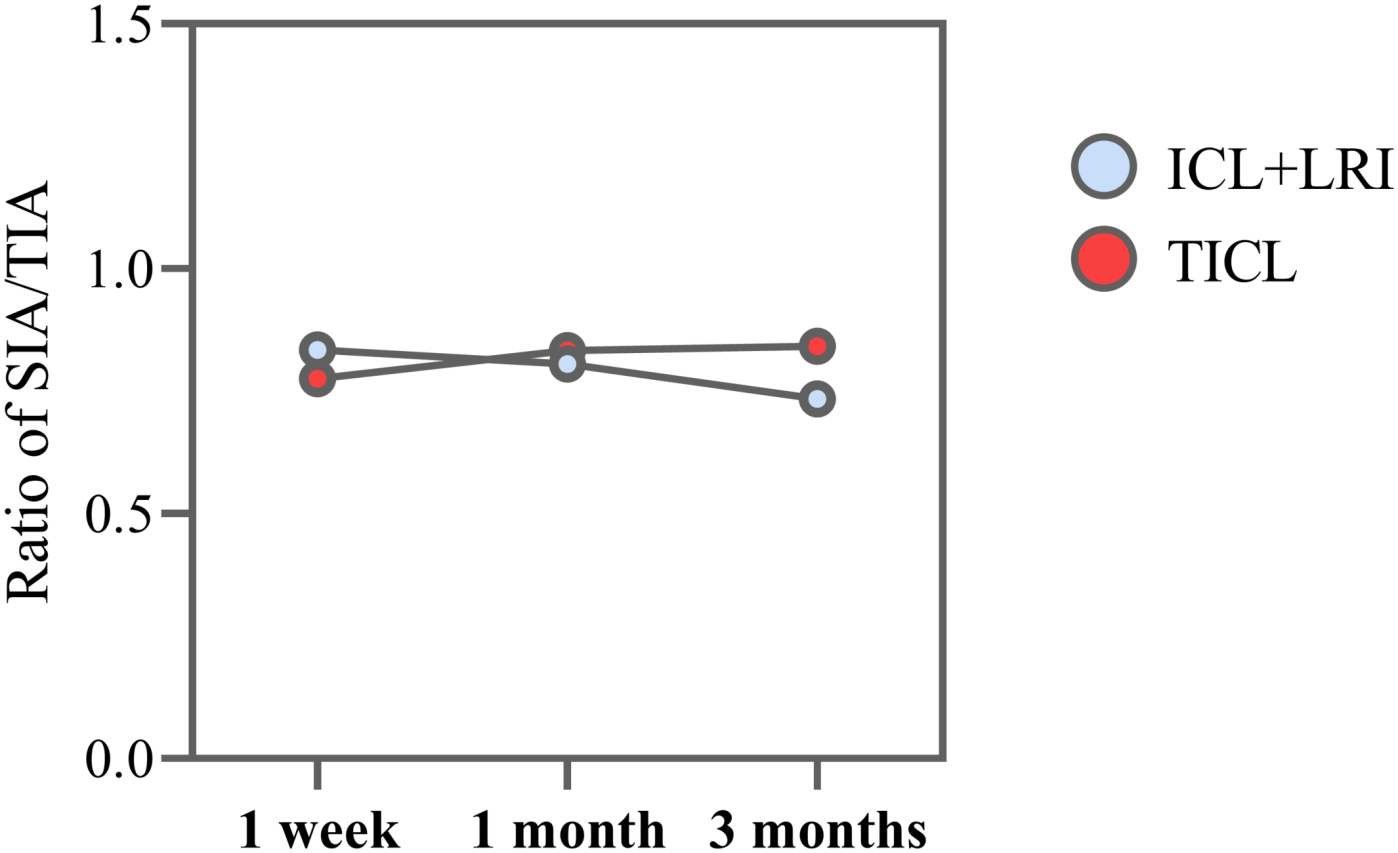
Postoperative correction index (CI) at different follow-up time points.

Changes in SIA and CI after surgery in the ICL + LRI and TICL groups are shown in **Table 2**, **Figures 3**, **4**. The SIA in the LRI group showed mild decreases, with a difference from 1 week to 3 months postoperatively (from 1.07 to 0.92, P = 0.044), while it remained stable in the TICL group (from 1.11 to 1.20, P = 0.507). A significant difference in SIA was observed between the two groups at 3 months postoperatively (P = 0.007). Additionally, the CI in the LRI group decreased numerically at 3 months (from 0.84 to 0.68, P = 0.097), while the CI in the TICL group remained stable at 3 months postoperatively (from 0.80 to 0.87, P = 0.349), with no significant difference between the groups (P = 0.097).

### 3.4 Postoperative vault height and topographic changes

At 3 months postoperatively, there were no statistically significant differences in vault height or anterior chamber depth between the two groups (**Table 2**). The change in anterior corneal astigmatism in the LRI group was significantly greater than that in the TICL group [LRI: -0.47 ± 0.41 vs TICL: 0.10 (IQR: -0.10,0.30), P< 0.001].

### 3.5 Postoperative changes in higher-order aberrations

Higher-order aberrations and their postoperative changes are shown in **Tables 3**, **4**. From the baseline, the anterior corneal HOAs, anterior corneal spherical aberration, and total corneal spherical aberration in the ICL + LRI group were higher than those in the TICL group. Postoperatively, the anterior corneal aberration, total corneal aberration, and 30° trefoil in the ICL + LRI group were lower than those in the TICL group. The LRI group exhibited reductions in anterior, posterior, and total corneal aberrations (TMAS), as well as anterior and total corneal horizontal coma, total corneal spherical aberration, while the TICL group showed slight increases in these parameters; the differences between the two groups were statistically significant, with all P-values< 0.05.

**Table 3:** Preoperative and postoperative aberration.

Aberration	Preoperative			Postoperative		
RMS	ICL+LRI	TICL	P value	ICL+LRI	TICL	P value
Anterior corneal surface						
TMAS	2.11 ± 0.47	2.20 ± 0.46	0.514	**1.73 ± 0.45**	**2.31 ± 0.45**	**< 0.001**
HOA	**0.45 ± 0.11**	**0.39 ± 0.07**	**0.037**	0.47 ± 0.12	0.43 ± 0.08	0.088
Trefoil 0°	-0.01 ± 0.10	0.03 ± 0.11	0.336	0.01 ± 0.10	0.04 (-0.03,0.08)	0.781
Trefoil 30°	0.01 ± 0.10	-0.00 ± 0.10	0.831	-0.00 ± 0.12	0.04 ± 0.13	0.123
Coma 0°	0.05 ± 0.16	0.05 ± 0.13	0.910	0.03 ± 0.17	0.06 ± 0.13	0.540
Coma 90°	-0.12 ± 0.24	-0.14 ± 0.17	0.704	-0.03 ± 0.25	-0.10 (-0.20,0.01)	0.118
Spherical aberration	**0.25 ± 0.09**	**0.18 ± 0.06**	**0.004**	0.25 ± 0.10	0.21 ± 0.06	0.082
Posterior corneal surface						
TMAS	0.92 ± 0.20	0.91 ± 0.13	0.974	0.87 ± 0.21	0.94 ± 0.13	0.163
HOA	0.19 ± 0.04	0.19 ± 0.03	0.943	0.19 (0.17,0.26)	0.20 ± 0.03	0.478
Trefoil 0°	0.00 ± 0.04	-0.01 ± 0.04	0.227	0.01 ± 0.10	-0.01 ± 0.03	0.543
Trefoil 30°	-0.02 ± 0.04	-0.03 ± 0.05	0.240	-0.00 ± 0.12	-0.03 ± 0.06	0.311
Coma 0°	-0.01 ± 0.03	-0.01 ± 0.03	0.887	0.03 ± 0.17	-0.01 ± 0.03	0.808
Coma 90°	-0.01 ± 0.04	-0.00 ± 0.04	0.496	-0.03 ± 0.25	-0.00 ± 0.05	0.281
Spherical aberration	-0.16 ± 0.03	-0.15 (-0.18, -0.14)	0.291	-0.16 ± 0.04	-0.16 ± 0.03	0.726
Total corneal						
TMAS	1.64 ± 0.43	1.71 ± 0.39	0.407	**1.36 ± 0.39**	**1.81 ± 0.37**	**< 0.001**
HOA	0.40 ± 0.10	0.35 ± 0.06	0.073	0.42 ± 0.11	0.37 ± 0.07	0.101
Trefoil 0°	-0.00 ± 0.10	0.02 ± 0.10	0.553	-0.00 ± 0.09	0.02 (-0.03,0.08)	0.823
Trefoil 30°	-0.00 ± 0.10	-0.03 ± 0.10	0.535	**-0.05 ± 0.10**	**0.02 ± 0.11**	**0.028**
Coma 0°	0.05 ± 0.14	0.05 ± 0.12	0.886	0.03 ± 0.16	0.05 ± 0.11	0.526
Coma 90°	-0.14 ± 0.19	-0.12 ± 0.15	0.887	-0.04 ± 0.21	-0.10(-0.17,-0.02)	0.145
Spherical aberration	**0.19 ± 0.08**	**0.13 ± 0.06**	**0.013**	0.18 ± 0.08	0.16 ± 0.06	0.299

RMS, root mean square; TMAS, the total Zernike root mean square; HOA, higher-order aberration.

Bold values indicate statistically significant differences (P < 0.05).

**Table 4:** Postoperative change in corneal aberration.

Aberration	Group		P value
RMS	LRI+ICL	TICL	
Anterior corneal surface			
TMAS	**-0.39 ± 0.47**	**0.11 ± 0.29**	**< 0.001**
HOA	0.03 ± 0.09	0.03 ± 0.05	0.617
Trefoil 0°	0.01 ± 0.13	-0.01 ± 0.11	0.538
Trefoil 30°	-0.02 ± 0.12	0.05 ± 0.12	0.068
Coma 0°	**-0.02 ± 0.05**	**0.01 ± 0.04**	**0.033**
Coma 90°	-0.02 ± 0.12	0.05 ± 0.12	0.076
Spherical aberration	0.00 ± 0.06	0.03 ± 0.04	0.080
Posterior corneal surface			
TMAS	**-0.05 ± 0.09**	**0.03 ± 0.11**	**0.019**
HOA	0.01 ± 0.02	0.01 ± 0.02	0.294
Trefoil 0°	-0.01 ± 0.05	-0.00 ± 0.05	0.814
Trefoil 30°	-0.00 ± 0.01	-0.00 ± 0.01	0.363
Coma 0°	-0.00 ± 0.01	0.00 ± 0.01	0.370
Coma 90°	-0.04 ± 0.05	-0.01 ± 0.06	0.051
Spherical aberration	-0.00 (-0.01,0.00)	0.00 (-0.01,0.01)	0.991
Total corneal			
TMAS	**-0.28 (-0.43, -0.12)**	**0.19 (-0.01,0.23)**	**0.001**
HOA	0.02 ± 0.09	0.02 ± 0.06	0.807
Trefoil 0°	0.01 (-0.08,0.06)	-0.03 (-0.08,0.06)	0.939
Trefoil 30°	-0.02 ± 0.06	0.01 ± 0.04	0.095
Coma 0°	**-0.02 ± 0.06**	**0.01 ± 0.04**	**0.048**
Coma 90°	0.05 (0.01,0.13)	0.02 (-0.08,0.06)	0.057
Spherical aberration	**-0.01 ± 0.07**	**0.03 ± 0.03**	**0.039**

RMS, root mean square; TMAS, the total Zernike root mean square; HOA, higher-order aberration.

Bold values indicate statistically significant differences (P < 0.05).

## 4 Discussion

To achieve optimal postoperative visual quality, various surgical modalities have been developed to manage astigmatism: CCIs (clear corneal incisions), LRIs, corneal laser surgery, arcuate keratotomies (AKs) and Toric implantable lens (13–15). TICLs show good stability in medium-to-long-term follow-ups, though refraction changes may still occasionally occur due to the lens rotation. Limbal relaxing incisions have advantages in flexibility of postoperative repositioning and economic perspective but carries the risk of astigmatism regression (16). there is currently no clear consensus regarding the preferred procedure in the treatment of moderate astigmatism (range from 1.00 to 2.00D). Therefore, to form a better decision-making framework, it is necessary to make a comprehensive evaluation of the correction efficacy, stability, and changes in aberrations of ICL + LRI and TICL implantation.

The present study showed that both groups achieved satisfactory visual and refractive outcomes, as well as good safety, consistent with the findings of Yang, Ke (9) et al. In our study, the IOP in the TICL group was higher than that in the LRI group at 1 week postoperatively, which may be related to the thicker preoperative central corneal thickness in the TICL group. This difference disappeared at 1 and 3 months postoperatively as the IOP stabilized, demonstrating favorable long-term IOP safety for both modalities.

In terms of vector analysis: The SIA and CI demonstrated reductions, while it remained stable in the TICL group, which is consistent with previous findings (9, 17). TICL implantation seems to have advantages in the stability of cylinder correction, although this variance was not reflected in the postoperative visual acuity or residual astigmatism.

Interestingly, the proportion of visual acuity not less than 1.0 is higher, and the proportion of UDVA lower than preoperative CDVA was lower in LRI group at 3 months in our study. A Meta-Analysis (8) indicated that in cataract surgeries Toric IOLs more likely to achieve astigmatism within 0.5 D compared with LRIs. But this difference is likely to be clinically unimportant. There was no evidence of an important difference in postoperative visual acuity or quality of life between the two techniques. We consider that the LRI group has a trend of astigmatism regression in the short term (3 months) postoperatively, but this change does not cause significant changes in visual acuity and refractive error.

Our study found that corneal astigmatism decreased by 0.47 ± 0.41D (35.18%) in LRI groups and the TICL group did not show significant difference, which is consistent with previous studies (11, 18, 19). The number of samples and the follow-up period could contribute to the variation in the reduction percentage and magnitude of corneal astigmatism.

In the analysis of corneal aberrations in this study, several corneal aberration parameters in the LRI group showed numerical reductions at 3 months postoperatively. A long-term follow-up study (11) found no significant changes in HOA after LRI, except trefoil (P = 0.004), the discrepancy in postoperative HOAs changes may be attributed to variations in ethnicities and the specific diagnostic platforms employed between the two studies. In terms of corneal refractive surgery, it has been demonstrated that HOAs change significantly following both ICL and SMILE surgeries (20), postoperative coma values of 1.17 ± 0.82 and 2.88 ± 2.14 (P< 0.001), and spherical aberration values of -1.43 ± 1.09 and -3.87 ± 2.43 (P< 0.001), respectively. These findings indicate that the ICL surgeries have milder effects on aberration changes compared to SMILE. The discrepancy between their reported HOAs and our results lies mainly in differences in the measurement devices (total ocular wavefront vs. corneal topography) and the selection of aberration orders (total HOAs vs. 3rd- and 4th-order aberrations). Combined with our findings on the divergence between the statistical decline of SIA (from 1.07 to 0.92 D) and the stability of clinical visual outcomes, The clinical significance of these HOA changes remains to be further validated by subjective and objective visual quality assessments.

Considering setting of the maximum analysis diameter, previous study (21) indicated that more than 99% of the RMS wave-front error is contained for a 5.00mm pupil diameter in Zernike Analysis. 4.00 and 6.00mm were commonly selected in previous studies as analysis diameter (22, 23). According to a foundational standard (24), a standardized 6.00mm pupil size is critical for achieving methodological uniformity and enabling direct comparisons across global refractive literature. Taking the effect of LRIs on mid-peripheral cornea into account, we think 6.00mm is sufficient as the analysis area.

This study has several limitations: It has a small sample size (30 patients, 46 eyes) and a short follow-up period (3 months), though previous study (19) indicated that effect of LRI was stable between 10 weeks to 3 years. Future studies can expand the sample size, extend the follow-up period and the range of preoperative cylinder. In addition, this study did not include subjective visual quality scores of patients, while subjective feelings are important references for surgical procedure selection. Relevant indicators can be supplemented in future studies to make the research conclusions more comprehensive.

## 5 Conclusion

Both ICL combined with LRI and TICL implantation achieve good efficacy in correcting moderate astigmatism. The TICL group showed better postoperative astigmatism stability. The LRI group demonstrated reductions in several corneal aberration parameters compared with the TICL group.

## Data Availability

The raw data supporting the conclusions of this article will be made available by the authors, without undue reservation.
